# Long-term visual outcomes and histopathologic findings after cataract surgery in Ebola virus disease survivors in the Ebola virus RNA persistence in ocular tissues and fluids (EVICT) study

**DOI:** 10.1371/journal.pntd.0012662

**Published:** 2024-11-22

**Authors:** Caleb D. Hartley, Lucas Kim, Tolulope Fashina, Jack Begley, John G. Mattia, Matthew J. Vandy, Lloyd C. Harrison-Williams, Jalikatu Mustapha, Robert F. Garry, John S. Schieffelin, Donald S. Grant, Augustine Goba, Colleen S. Kraft, Brent R. Hayek, Gustavo Palacios, Jessica Shantha, Ian Crozier, Xiankun Zeng, Steven Yeh, Ebola Virus Persistence in Ocular Tissues and Fluids (EVICT) Study Investigators

**Affiliations:** 1 Truhlsen Eye Institute, Department of Ophthalmology, University of Nebraska Medical Center, Omaha, Nebraska, United States of America; 2 National Eye Programme, Ministry of Health and Sanitation, Freetown, Sierra Leone; 3 Tulane University School of Medicine, Tulane University, New Orleans, Louisiana, United States of America; 4 Kenema Government Hospital, Lassa Hemorrhagic Fever Laboratory, Kenema, Sierra Leone; 5 Department of Community Health, University of Sierra Leone, Freetown, Sierra Leone; 6 Emory University School of Medicine, Emory University, Atlanta, Georgia, United States of America; 7 North Georgia Eye Associates, Gainesville, Georgia, United States of America; 8 Department of Microbiology, Icahn School of Medicine at Mt. Sinai, New York, New York State, United States of America; 9 Francis I. Proctor Foundation for Ophthalmic Research, University of California San Francisco, San Francisco, California, United States of America; 10 Clinical Monitoring Research Program Directorate, Frederick National Laboratory for Cancer Research, Frederick, Maryland, United States of America; 11 United States Army Medical Research Institute of Infectious Diseases, Fort Detrick, Maryland, United States of America; 12 Global Center for Health Security, University of Nebraska Medical Center, Omaha, Nebraska, United States of America; Hokkaido University Research Center for Zoonosis Control, JAPAN

## Abstract

**Background/Objectives:**

Ebola virus disease (EVD) survivors develop post-acute ophthalmic sequelae, including a high prevalence of uveitis that may be complicated by vision-threatening cataract. After the non-detection of Ebola virus (EBOV) RNA in sampled ocular fluid, vision impairment due to cataract can be treated safely and effectively via manual small incision cataract surgery (MSICS). However, the long-term ocular visual outcomes and assessment of ocular tissues, including for genomic RNA, have been infrequently or not reported in Western African survivors.

**Subjects/Methods:**

A cohort of EVD survivors with visually significant cataract, in whom EBOV RNA was not detected by RT-PCR testing of ocular fluid sampled prior to MSICS, were followed for a year after intraocular lens replacement. Ophthalmic outcomes, including visual acuity (VA), adverse events, and follow-up examinations, were recorded. Ocular tissue specimens obtained at MSICS underwent histopathologic examination and in-situ hybridization (ISH) targeting EBOV genomic RNA.

**Results:**

Thirty-four EVD survivors underwent MSICS and were included for analysis. The median age of EVD survivors who underwent surgery at enrollment was 22.5 years (Interquartile range, IQR: 16.5–33) and the cohort was comprised of 20 females (58.8%). Median logMAR VA at preoperative baseline was 3 (IQR: 1–3) which improved to 0.4 (IQR: 0–0.6; n = 10; p = 0.002) and 0.6 (IQR: 0.18–0.78; n = 18; p < 0.0001) at 6- and 12-months following surgery, respectively. EBOV RNA was not detected in 7 cataract and capsular tissue specimens obtained at the time of MSICS.

**Conclusions:**

After MSICS, meaningful improvement in vision was maintained in EVD survivors at long-term follow-up. EBOV RNA was not detected in cataract and lens capsule specimens, providing additional reassurance of the low risk of EBOV RNA exposure during cataract surgery. Further study is needed to understand long-term ocular outcomes, including adverse events, in this population.

**Author summary:**

Ebola virus disease (EVD) survivors may develop several ophthalmic sequelae including uveitis and cataract that may lead to severe vision loss if left untreated. Manual small incision cataract surgery (MSICS) has been utilized to treat cataract in EVD survivors with encouraging short-term outcomes, but the long-term visual acuity outcomes and the potential for Ebola virus RNA to reside in cataract material is unknown. In this study, we reported long-term visual acuity outcomes following MSICS in a cohort of EVD survivors along with histopathology findings from materials collected during surgery. Visual acuity improved over 12-month follow-up with encouraging safety measures. Ebola virus RNA was not detected in cataract and lens capsular tissue analyzed from EVD survivors. This study provides additional assurance regarding the safety and efficacy of cataract surgery, with potential for improved vision for EVD survivors.

## Introduction

The 2013–2016 outbreak of Ebola virus disease (EVD) in Western Africa was the largest outbreak to-date, leading to over 11,000 deaths and nearly 28,600 documented cases [1]. Since the outbreak, significant efforts have been made to understand previously underrecognized post-acute sequelae of EVD, including a high prevalence of ophthalmic disease in survivors [2–8]. While sight-threatening uveitis (inflammation of the eye) [2,3,8], cataracts (clouding of the lens of the eye) [2,3,8], and optic neuropathy (damage to the optic nerve) [9] have been clinically described, the long-term visual outcomes from EVD in survivors have been less frequently reported [3,8]. Histopathologic characterization, including examination of ocular tissues for Ebola virus (EBOV) genomic RNA, has not been reported from Western Africa.

Infectious EBOV has been previously detected in the aqueous humor of an EVD survivor with severe post-acute panuveitis who subsequently developed cataracts following resolution of uveitis. Persistent EBOV RNA has been identified in ocular tissues or fluids of non-human primate survivors, including in association with clinical uveitis and destructive intraocular histopathologic abnormality [5,10,11]. Prospective studies of EVD survivors in Sierra Leone and Liberia who were anticipating manual small incision cataract surgery (MSICS) assessed EBOV RNA in preoperatively sampled ocular fluid by reverse-transcriptase polymerase chain reaction (RT-PCR) [3,8]. In both studies, EBOV RNA was not detected in aqueous humor and EVD survivors were subsequently able to undergo intraocular lens replacement to improve vision.

Molecular methods such as in-situ hybridization (ISH) have been used to detect EBOV RNA in the intraocular tissue of rhesus monkeys, notably in the vitreous humor, the retina, and the ciliary body of the posterior eye [10,11]. Similar examination for genomic RNA as well as general histopathologic characterization of anterior capsular tissue or lens material from Western African EVD survivors has not been reported.

To further understand long-term clinical outcomes following cataract surgery in EVD survivors, we reviewed the 12-month results following MSICS within the **E**bola **Vi**rus Persistence in O**c**ular **T**issues and Fluids (EVICT) study conducted in Sierra Leone. We also report the histopathological findings and ISH assessments of ocular tissue in a subset of patients who underwent further laboratory investigation.

## Methods

### Ethics statement

This study was approved by Emory University’s Institutional Review Board as well as the Office of Ethics and Scientific Review Committee, Sierra Leone Ministry of Health and Sanitation. Written informed consent was obtained prior to initiation of the study and associated study procedures. All activities were conducted according to the tenets of the Declaration of Helsinki.

Patients were identified by a Sierra Leone Ministry of Health and Sanitation National Eye Care Program from March 2015 through the end of March 2016. Specifically, EVD survivors who anticipated cataract surgery and patients with active uveitis where EBOV persistence was a potential concern were referred from Lunsar Baptist Eye Hospital (Port Loko), Kenema Government Hospital (Kenema), Connaught Government Hospital (Freetown), and the Sierra Leone Association of Ebola Survivors (SLAES) to Lowell and Ruth Gess Eye Hospital in Freetown for ophthalmologic examination and study enrollment [8]. Those who met eligibility criteria and elected to enroll were longitudinally followed and were included in this secondary data analysis.

Vision screening, ophthalmic examination, laboratory, and surgical procedures were conducted as reported previously [8]. Patients underwent an anterior chamber paracentesis for Ebola virus RNA detection under strict infection prevention and control guidance and monitoring by a clinician trained in precautions for high-consequence pathogens. Following a negative Ebola virus real-time reverse transcriptase PCR, patients were then eligible for the MSICS procedure [8].

After MSICS, patients were evaluated at postoperative day 1 and week 1. Patients were also assessed at month 1, 3–4, 6, and 12, or sooner as needed (S1 Fig). The visual acuity (VA) and ophthalmic findings, including slit lamp examination, were documented and recorded. Adverse events including recurrent uveitis, elevated intraocular pressure, new or worsening posterior capsular opacification, or other events of medical significance were recorded.

### Histopathology and RNA in situ hybridization

Ocular specimens including cataract material (i.e., removed en bloc as typical for MSICS) and lens capsules were stored in 10% neutral buffered formalin. The specimens were embedded in paraffin, and tissue sections were cut, deparaffinized, and stained with hematoxylin and eosin (H&E) using standard procedure. To detect EBOV genomic RNA in tissue specimens, RNA *in situ* hybridization (ISH) was performed using the RNAscope 2.5 HD Detection Kit (RED) for FFPE Tissues (Advanced Cell Diagnostics, Newark, CA, USA) according to the manufacturer’s instructions. Briefly, an ISH probe targeting the genomic fragment 1673–2598 of EBOV nucleoprotein (NP) gene with GenBank no. J04337.1 was designed and synthesized by Advanced Cell Diagnostics (Cat# 448581). Tissue sections were deparaffinized with Xyless II (Val Tech Diagnostics, Brackenridge, PA, USA), underwent a series of ethanol washes and peroxidase blocking, heated in kit-provided antigen retrieval buffer, and then digested by kit-provided proteinase. Sections were exposed to ISH target probes and incubated at 40°C in a hybridization oven for 2 hours. After rinsing, ISH signal was amplified using kit-provided pre-amplifier and amplifier conjugated to alkaline phosphatase and incubated with a Fast Red substrate solution for 10 min at room temperature. Sections were then stained with hematoxylin, air-dried, and cover slipped.

### Statistical analyses

Snellen VA values were converted to logMAR for analytic purposes and hand motions and counting fingers measurements were handled as described previously [12]. Wilcoxon signed-rank tests were employed to evaluate differences in VA between baseline and a given postoperative timepoint following MSICS [13]. Statistical analyses were conducted using α = 0.05 as the threshold for statistical significance and with SAS statistical software (SAS Institute, Cary, NC, USA).

## Results

Of one hundred-thirty-seven EVD survivors from Sierra Leone screened between June 2016 and August 2017, 50 subjects were enrolled in the EVICT study. Thirty-four survivors underwent MSICS and were included for baseline and follow-up assessment. Patient characteristics, including VA, were previously reported at pre-operative and post-operative (1 months and 3/4 month) timepoints [8]. Twenty women (58.8%) made up the 34-person cohort and median age at enrollment for the cohort was 22.5 years (IQR: 16.5–33).

**Fig 1:**
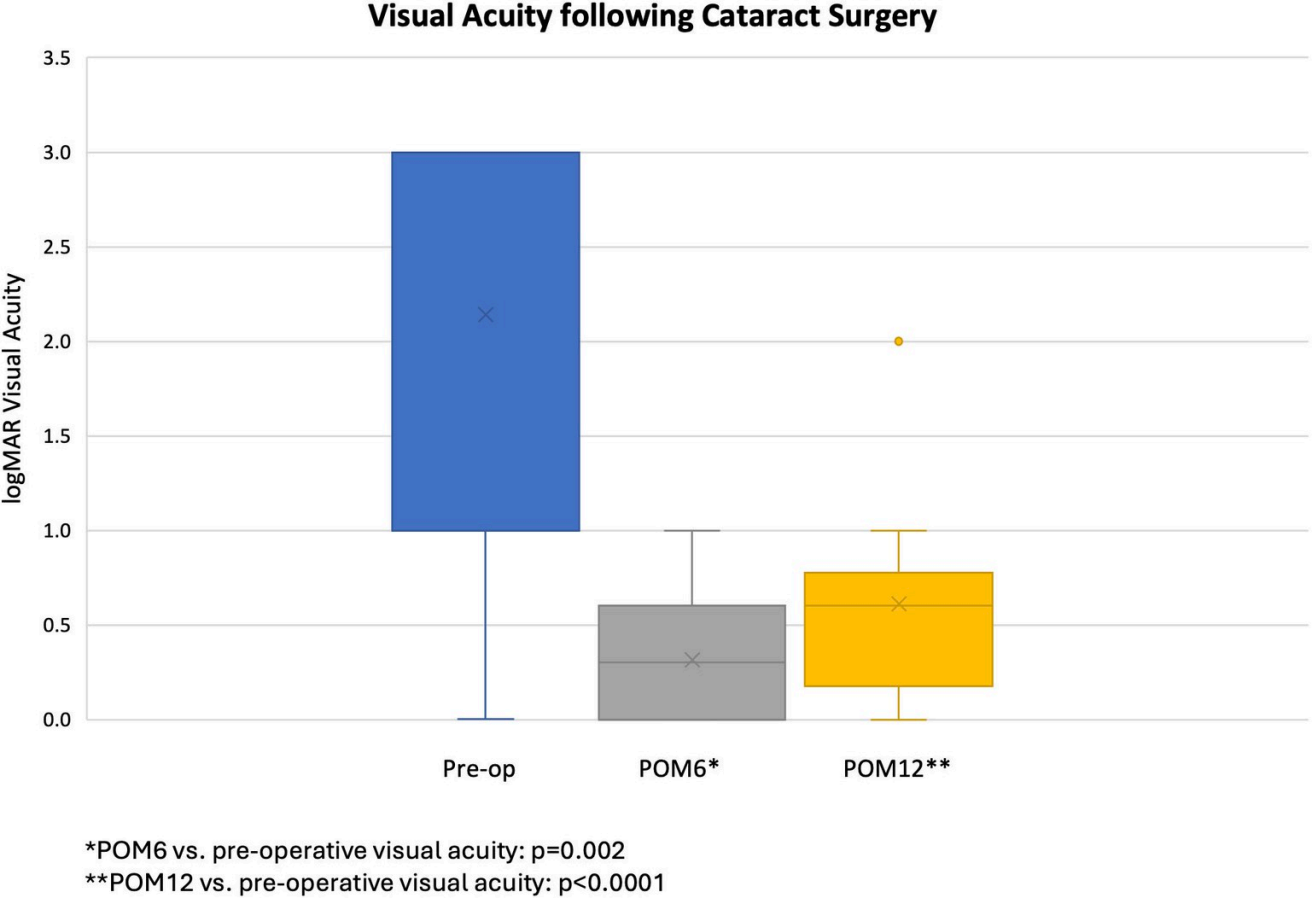
Visual acuity (VA) in eyes after cataract surgery. Preoperative median logMAR VA was 3.0 (Snellen VA equivalent: hand motions) with improvement to 0.4 (Snellen VA equivalent: 20/50) at 6 months following surgery (n = 10). Median VA at 12 months worsened to 0.6 (Snellen VA equivalent: 20/80; n = 18).

Compared to the preoperative median logMAR VA of 3 (interquartile range (IQR: 1–3) [8], vision improved to 0.4 (IQR: 0–0.6) at 6 months after surgery (n = 10; p = 0.002). Compared to the preoperative logMAR VA, vision improved to 0.6 (IQR: 0.18–0.78) at 12 months following MSICS (n = 18; p < 0.0001) (Fig 1). These logMAR values correspond to a preoperative median Snellen VA of hand motions (IQR: 20/200 –hand motions) which improved to 20/50 (IQR: 20/20-20/80) at 6 months after surgery and 20/80 (IQR: 20/30-20/120) at 12 months following surgery.

Five of 18 patients (28%) who followed-up at 12 months, showed a Snellen VA of 20/40 or better while 11 of 18 patients (62%) demonstrated Snellen VA between 20/50 and 20/200.

### Ocular adverse events

Ocular adverse events were recorded at each ophthalmic examination following MSICS and included both anterior and posterior conditions (Table 1). No ocular adverse events were noted in more than 40% of patients during follow-up. Posterior capsular opacification, observed in 26% of patients, was the most commonly encountered adverse event during the postoperative period. Less commonly, recurrence of uveitis (anterior chamber inflammation prompting anti-inflammatory treatment) and vitreous opacity was noted in 21% and 9% of patients during follow-up, respectively. Fibrotic plaque, fibrotic membrane, epiretinal membrane, and tractional retinal detachment were noted in one patient.

**Table 1:** Characteristics of patients who underwent Manual Small Incision Cataract Surgery (MSICS).

**Demographic characteristics**			
Age, Median, (IQR), years	22.5 (16.5–33)		
Total no. of patients Women, n (%) Men, n (%)	34 20 (58.8%) 14 (41.2%)		
**Snellen Visual Acuity Outcomes**	Baseline*	6 months**	12 months***
20/40 or better, n (%) 20/50-20/200, n (%) 20/200-20/400, n (%) VA < 20/400, n (%)	3 (8.8%) 2 (5.9%) 6 (17.7%) 23 (67.6%)	5 (50%) 5 (50%) 0 0	5 (27. 8%) 11 (61.2%) 1 (5.6%) 1 (5.6%)
**Safety Outcomes**			
No observed complications, n (%) Posterior capsular opacification, n (%) Uveitis recurrence, n (%) Vitreous opacity, n (%) Fibrotic plaque, n (%) Fibrotic membrane, n (%) Epiretinal membrane, n (%) Traction retinal detachment, n (%)	15 (44.1%) 9 (26%) 7 (20.6%) 3 (8.8%) 1 (2.9%) 1 (2.9%) 1 (2.9%) 1 (2.9%)		

† IQR: interquartile range; VA: visual acuity

*n = 34

**n = 10

***n = 18

### Histopathology and in situ hybridization for EBOV RNA

**Fig 2:**
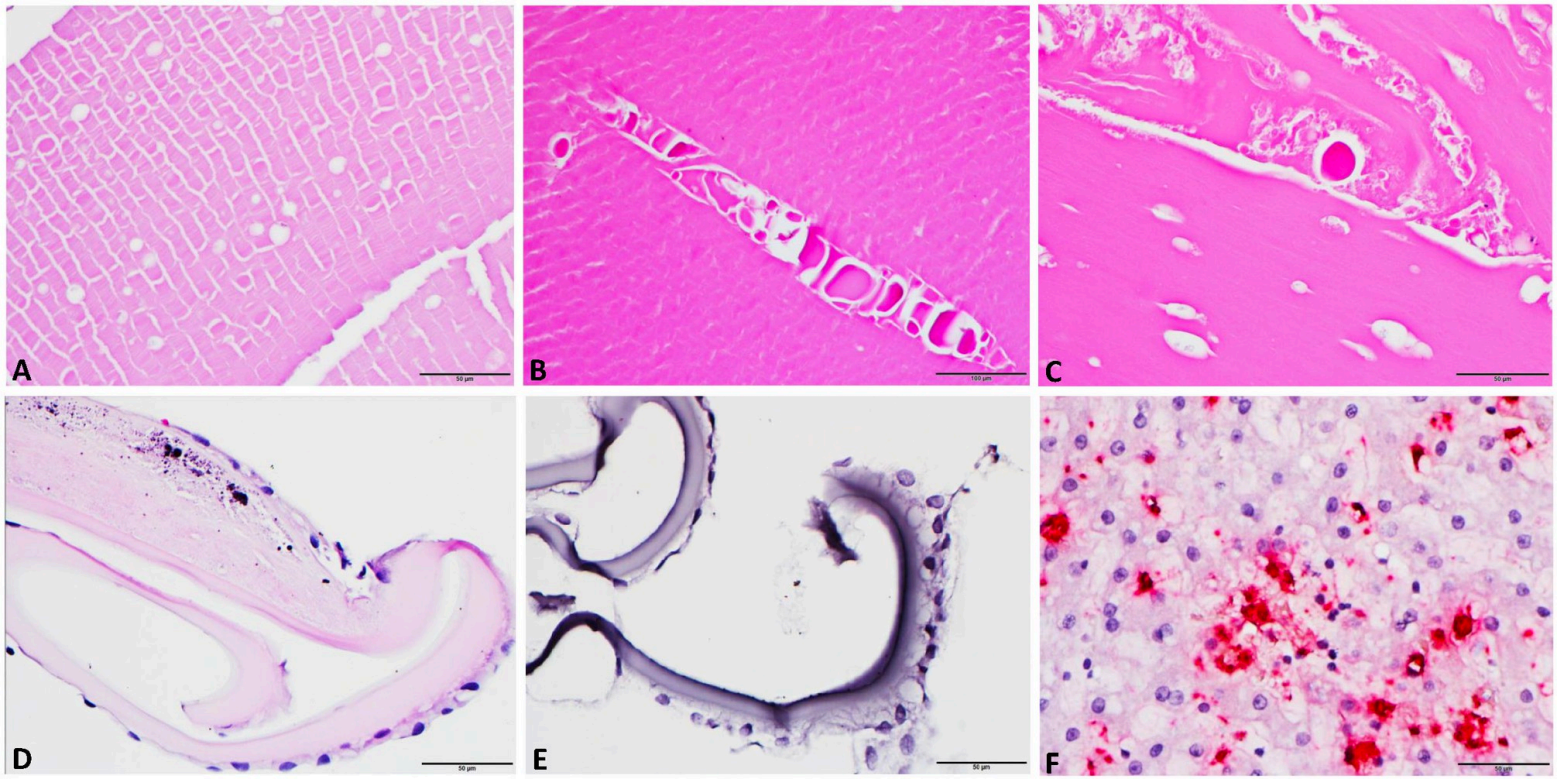
Histopathologic features of and Ebola virus RNA detection by in situ hybridization (ISH) for anterior capsule and cataract tissues obtained during manual small incision cataract surgery (MSICS) from Ebola virus disease survivors. (A) Histologic section of cataract material shows lens fiber vacuoles. (B) Morgagnian globules and clefts in cataract fragments are also observed. (C) A higher-magnification view highlights vacuoles within lens fibers and Morgagnian globules. (D) Mineralization of the anterior capsular and capsular thickening are observed, which corresponds to fibrosis and thickening of the lens capsules of EVD survivors with prior uveitis. (E) ISH, representative sample: EBOV nucleoprotein (NP) genomic RNA is not detected. (F) ISH: EBOV-NP genomic RNA detection in liver tissue of an infected rhesus monkey (positive control).

Lens and capsular materials collected from seven patients during MSICS were histologically examined and analyzed for EBOV RNA by ISH. Histopathology of cataracts and cataract nuclei showed lens fiber vacuolation and Morgagnian globules; mineralization and a thickened capsule were noted in anterior lens capsules (n = 2). By ISH, EBOV genomic RNA was not detected in lens or capsular material of seven lens samples (Fig 2).

## Discussion

Our prior work demonstrated that MSICS, following the non-detection of EBOV RNA in aqueous humor, can effectively and safely restore vision in EVD survivors with cataract [3,8]. In this evaluation of the long-term efficacy and safety outcomes, improved VA in patients who returned for 12-month follow-up after MSICS was sustained, although there was a subtle reduction in VA in a subset of patients from 3–4 month to 6- and 12-month post-operative timepoints. The pathophysiology and risk factors for this decline warrant further investigation.

In this Sierra Leonean population, the median preoperative VA was worse at presentation than in Liberian EVD survivors similarly characterized before and after MSICS in the Partnership for Research on Ebola Virus in Liberia (PREVAIL) VII study [3]. In both the PREVAIL VII study and this analysis, VA of EVD survivors significantly improved overall from the preoperative to 12-month postoperative timepoints. Ocular adverse events observed during our follow-up included recurrent uveitis, vitreous opacity and fibrosis, posterior capsular opacification, and epiretinal membrane. A modest proportion of EVD survivors in our study developed posterior capsular opacification that required YAG capsulotomy, which was similar to findings from the PREVAIL VII study [3].

A concern related to cataract surgery after EVD is the potential risk posed by EBOV persistence in ocular fluids and tissues. We previously reported the non-detection of EBOV RNA in the sampled intraocular fluid (predominantly aqueous humor) of 50 EVD survivors enrolled into the EVICT study; similar findings were confirmed in 22 EVD survivors who underwent MSICS in the PREVAIL VII. In general, histopathologic findings in the lens and capsular materials from EVD survivors with cataracts were unsurprising, and the non-detection of EBOV RNA by ISH of anterior segment ocular tissues harvested at the time of MSICS was encouraging.

This study was limited by patient adherence to longitudinal follow-up made challenging by logistical barriers, including the financial burden of travel for patients coming from rural districts to the capital city of Freetown, Sierra Leone for evaluation. Additionally, the 6- and 12-month outcomes were not pre-specified endpoints for primary outcome analysis. Finally, ophthalmic assessment reporting was limited as visual acuity documentation was not necessarily best-corrected visual acuity with a manifest refraction. It also should be noted that the sensitivity of ISH targeting genomic EBOV-NP in intraocular tissues is undetermined.

Despite these limitations, our longer-term assessment in Sierra Leonean EVD survivors with visually significant cataracts showed that improvement in VA was sustained after MSICS. While a subtle decrease in VA was observed in a subset of patients between months 6 and 12, these changes could be explained by posterior capsular opacification, recurrent uveitis, and vitreous opacity, which are treatable conditions. Our findings emphasize the importance of longer-term follow-up and the need to understand the potential for ocular adverse events and prompt treatment during follow-up. In addition, the histopathologic and molecular pathologic assessments are the first reports from West African survivors and provide additional assurance to ophthalmic surgeons of the likely low risk of EBOV RNA exposure prior to and during cataract surgery for EVD survivors. Further studies to understand the long-term outcomes following cataract surgery in EVD survivors are needed to better inform clinical guidance for patients, as well as safety considerations for the ophthalmic health care provider team.

## Supporting information

S1 Fig Flow chart summarizing patient enrollment, screening, treatment, and follow-up of Ebola virus disease (EVD) survivors including those who underwent manual small incision cataract surgery (MSICS) in the Ebola Virus Persistence in Ocular Tissues and Fluids (EVICT) study. (TIFF)

S1 Data Visual acuity data and long-term outcomes following cataract surgery in EVD survivors. (XLSX)
